# CD8/CD45RO T-cell infiltration in endoscopic biopsies of colorectal cancer predicts nodal metastasis and survival

**DOI:** 10.1186/1479-5876-12-81

**Published:** 2014-03-29

**Authors:** Viktor H Koelzer, Alessandro Lugli, Heather Dawson, Marion Hädrich, Martin D Berger, Markus Borner, Makhmudbek Mallaev, José A Galván, Jennifer Amsler, Beat Schnüriger, Inti Zlobec, Daniel Inderbitzin

**Affiliations:** 1 Translational Research Unit (TRU), Institute of Pathology, University of Bern, Murtenstr. 31, Bern CH-3010, Switzerland; 2 Clinical Pathology Division, Institute of Pathology, University of Bern, Bern, Switzerland; 3 Departments of Visceral Surgery and Medicine, Bern University Hospital, Bern, Switzerland; 4 Department of Surgery, Tiefenau Hospital, Bern, Switzerland; 5 Department of Medical Oncology, Bern University Hospital, Bern, Switzerland; 6 Department of Oncology, Hospital Centre Biel, Biel, Switzerland

**Keywords:** Pre-operative biopsies, Colorectal cancer, Pre-operative risk stratification, Immune infiltration, Prognostic factor, Nodal metastasis

## Abstract

**Background and aims:**

Reliable prognostic markers based on biopsy specimens of colorectal cancer (CRC) are currently missing. We hypothesize that assessment of T-cell infiltration in biopsies of CRC may predict patient survival and TNM-stage before surgery.

**Methods:**

Pre-operative biopsies and matched resection specimens from 130 CRC patients treated from 2002-2011 were included in this study. Whole tissue sections of biopsy material and primary tumors were immunostained for pancytokeratin and CD8 or CD45RO. Stromal (s) and intraepithelial (i) T-cell infiltrates were analyzed for prediction of patient survival as well as clinical and pathological TNM-stage of the primary tumor.

**Results:**

CD8 T-cell infiltration in the preoperative biopsy was significantly associated with favorable overall survival (CD8i p = 0.0026; CD8s p = 0.0053) in patients with primary CRC independently of TNM-stage and postoperative therapy (HR [CD8i] = 0.55 (95% CI: 0.36-0.82), p = 0.0038; HR [CD8s] = 0.72 (95% CI: 0.57-0.9), p = 0.0049). High numbers of CD8i in the biopsy predicted earlier pT-stage (p < 0.0001) as well as absence of nodal metastasis (p = 0.0015), tumor deposits (p = 0.0117), lymphatic (p = 0.008) and venous invasion (p = 0.0433) in the primary tumor. Infiltration by CD45ROs cells was independently associated with longer survival (HR = 0.76 (95% CI: 0.61-0.96), p = 0.0231) and predicted absence of venous invasion (p = 0.0025). CD8 counts were positively correlated between biopsies and the primary tumor (r = 0.42; p < 0.0001) and were reproducible between observers (ICC [CD8i] = 0.95, ICC [CD8s] = 0.75). For CD45RO, reproducibility was poor to moderate (ICC [CD45i] = 0.16, ICC [CD45s] = 0.49) and correlation with immune infiltration in the primary tumor was fair and non-significant (r[CD45s] = 0.16; p = 0.2864). For both markers, no significant relationship was observed with radiographic T-stage, N-stage or M-stage, indicating that assessment of T-cells in biopsy material can add additional information to clinical staging in the pre-operative setting.

**Conclusions:**

T-cell infiltration in pre-operative biopsy specimens of CRC is an independent favorable prognostic factor and strongly correlates with absence of nodal metastasis in the resection specimen. Quantification of CD8i is highly reproducible and allows superior prediction of clinicopathological features as compared to CD45RO. The assessment of CD8i infiltration in biopsies is recommended for prospective investigation.

## Background

In solid cancers, the tumor microenvironment represents the interface between tumor, stromal and immune cells. In 2011 Hanahan and Weinberg defined the active evasion of cancer cells from attack and elimination by immune cells as a newly emerging hallmark of cancer [1]. One of the major challenges in cancer research is the translation of knowledge from basic science into daily diagnostic practice. In colorectal cancer (CRC), immunohistochemical double-staining allows an optimal visualization of the tumor-microenvironment by using a pancytokeratin marker for tumor cells and CD8 and CD45RO for T-cell subtypes [2,3]. Indeed, cancer cells involved in epithelial-mesenchymal transition (EMT) are also known in the literature as “tumor buds” or “EMT-like tumor cells” and represent a morphological parameter of tumor progression and worse survival [4-8]. In their surroundings, T-cells are the most frequently detected immune cell type with a strong evidence-based prognostic power [3,9-12].

The tumor microenvironment is a tumor area that is not only found at the invasive front of CRC, but also within the main tumor body. Consequently, the microenvironment containing intra-tumoral buds (ITB) and T-cells can also be sampled in superficial tumor regions in the preoperative biopsy. Importantly, the presence of ITB in biopsies of colon and rectal cancer patients has recently been associated with local and distant metastases [13-15]. This approach has important clinical consequences as it allows better prediction of cancer biology already in the preoperative setting and significantly extends the information that can be obtained from biopsy material.

Assessment of biomarkers on biopsy material may also provide additional independent prognostic and predictive information [16]. This approach may thus aid standard radiographic and clinical staging of the CRC patient before surgery. Consequently, we aim to address whether the presence of CD8 and CD45RO positive T-cells in preoperative biopsies of colon and rectal cancer is predictive of patient survival and TNM-stage thus providing optimal support to oncologists, gastroenterologists and surgeons in the preoperative patient management.

## Methods

### Patients

346 primary CRC patients surgically treated from 2002-2011 at the Insel University Hospital (Bern, Switzerland) were initially included in this study. A matched preoperative diagnostic biopsy was available for 185 cases. An experienced gastrointestinal pathologist (AL) and two residents (VHK and HD), blinded to the findings from the preoperative biopsy reviewed all Haematoxylin and Eosin (HE) slides from surgical resections. All resection specimens were classified according to the TNM 7th edition. Presence of lymphatic invasion (L), venous invasion (V), perineural invasion (Pn), tumor grade (G), number of tumor deposits, histological subtype and number of lymph nodes harvested was recorded. Tumor regression grade for patients preoperatively treated with radio and/or chemotherapy was graded according to recommendations by the College of American Pathologists. Clinical data were obtained from patient records including age at diagnosis, gender, tumor location, clinical TNM-staging, and information on preoperative or postoperative therapy. Endpoints of interest were TNM-stage and overall survival. Information on overall survival was available for 76 patients (median survival 73.5 months, 95% CI: 29-105). 30 patients were treated pre-operatively and 63 received adjuvant therapy. All preoperative biopsies were re-reviewed. Information on the number of biopsies per patient and the number of biopsies containing tumor was determined.

### Assay methods

Patient material was fixed in 10% buffered formalin and paraffin-embedded at the Institute of Pathology, University of Bern, Switzerland. Tumor blocks were sectioned at 4 μm. An immunohistochemistry double-stain for pancytokeratin marker AE1/AE3 (Dako, mouse monoclonal, 1:200, enzyme pre-treatment 5 minutes; DAB chromogen) and CD8 (Dako C8/144B, 1:100, pre-treatment with Tris buffer, at 95° for 20 minutes; AEC chromogen) as well as for AE1/AE3 and CD45RO (Abcam, 1:4000; pre-treatment with citrate buffer, at 100° for 30 minutes; AEC chromogen) using an automated Leica Bond III instrument with Haematoxylin counterstaining was performed. The local ethics committee of the Insel University Hospital (16-03-12) approved this study.

### Evaluation of immunohistochemistry

**Figure 1:**
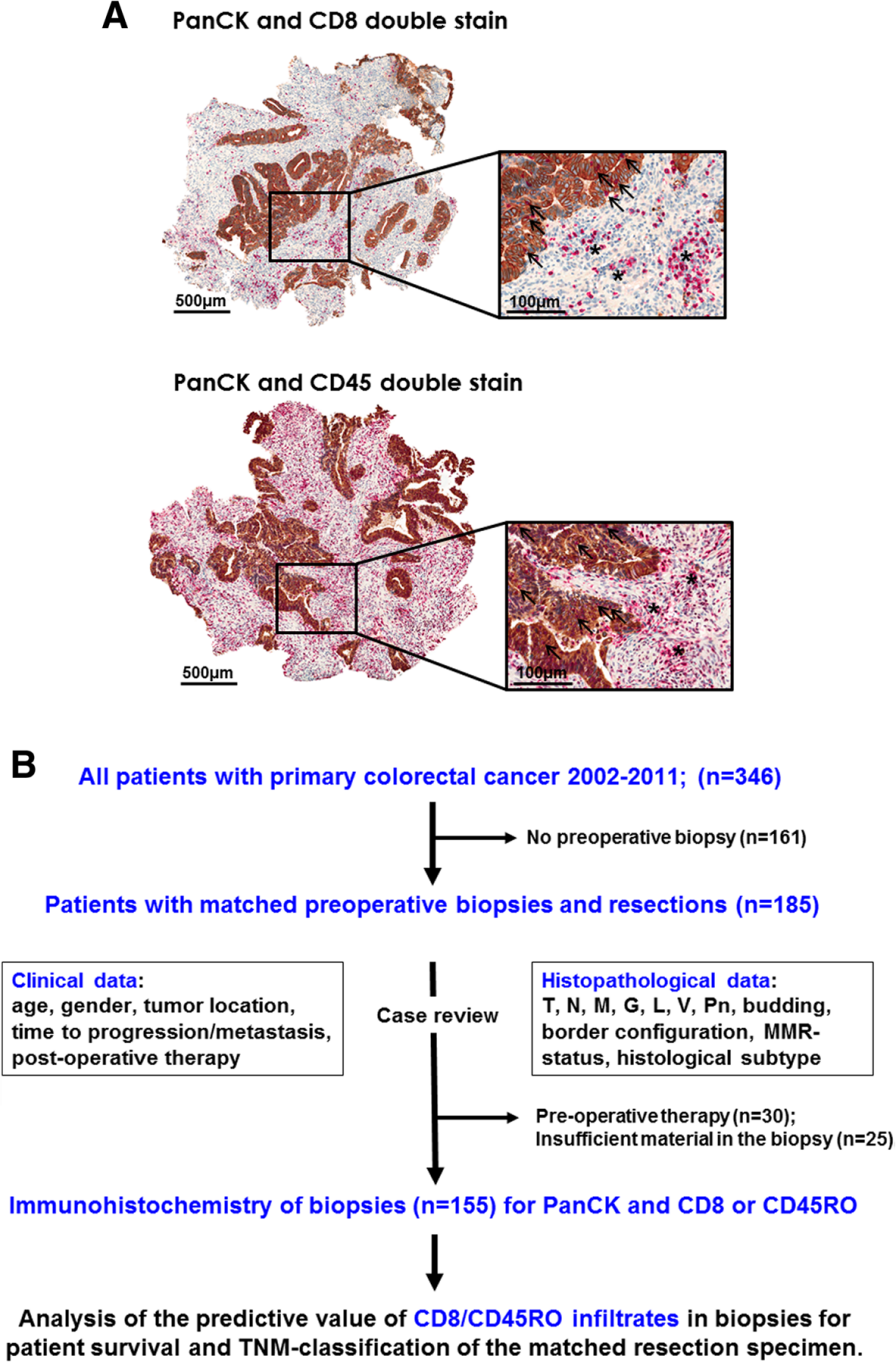
**Visualization of immune infiltrates in biopsy material and study design. A:** Visualization of immune infiltrates and tumor cells by immunohistochemical double-stain for CD8 (upper panel) or CD45RO (lower panel) and pancytokeratin in biopsy material (overview x 50, inset x 300). Stromal infiltrates are indicated by *, arrows indicate intraepithelial CD8+ or CD45RO+ T-cells. **B:** Study Design. 346 CRC patients were entered into the study, 185 of which had matched biopsies. Histopathological features and clinical data were re-reviewed. Immunohistochemistry for pancytokeratin and CD8 or CD45RO was performed. CD8+ and CD45RO+ T-cell infiltrates in biopsy specimens were evaluated in five HPF each of stroma and neoplastic epithelium. T-cell infiltrates were analysed for prediction of patient survival and histopathologic features of the matched resection specimen.

All biopsies from a single patient were first scanned at low-power magnification (10x) and the five densest regions of intratumoral (i) and stromal (s) CD8+ and CD45RO+ T-cells were identified (Figure 1A). Within these areas, CD8i, CD8s, CD45ROi, and CD45ROs T-cells were then counted in 5 high-power fields each (HPF; 40x; area 0.1886 mm^2^). The average number of infiltrating T-cells per HPF was determined for each area and used for statistical analysis.

### Study design

This study was designed according to the REMARK guidelines for tumor marker prognostic studies [17]. The study design is outlined in (Figure 1B). Of the 346 patients initially identified with surgically treated primary CRC from 2002-2011, 185 had matched preoperative biopsies. Patients receiving preoperative therapy (n = 30) were excluded from further analysis based on their small number. Immunohistochemistry was performed on the remaining 155 preoperative biopsies. Twenty-five cases had to be excluded from the study due to insufficient invasive carcinoma remaining in the diagnostic biopsy block. The final number of patients was 130. Patient characteristics are found in (Table 1).

**Table 1:** Patient characteristics and association of CD8+ cells (intra- and peri-tumoral) with clinicopathological data (max n = 130)

**Feature**			**CD8i N (%)**		**CD8s N (%)**			
		**N (%)**	**Low**	**High**	**P-value**	**Low**	**High**	**P-value**
Age (years) (n = 130)	Median (min, max)	72 (30-91)	71.4 (30-91)	73.8 (50-90)	0.3872	72.4 (30-91)	71 (30-90)	0.876
Gender (n = 130)	Male	80 (61.5)	61 (61.0)	19 (63.3)	1.0	40 (61.5)	40 (61.5)	1.0
	Female	50 (38.5)	39 (39.0)	11 (36.7)		25 (38.5)	25 (38.5)	
Histological subtype (n = 130)	Non-mucinous	106 (81.5)	80 (80.0)	26 (86.7)	0.5923	49 (75.4)	57 (87.7)	0.1123
	Mucinous	24 (18.5)	20 (20.0)	4 (13.3)		16 (24.6)	8 (12.3)	
Tumour location (n = 129)	Left	43 (33.3)	33 (33.3)	10 (33.3)	0.8606	21 (32.3)	22 (34.4)	0.7333
	Rectum	30 (23.3)	22 (22.2)	8 (26.7)		17 (26.2)	13 (20.3)	
	Right	56 (43.4)	44 (44.4)	12 (40.0)		27 (41.5)	29 (45.3)	
pT (n = 130)	pT1 + pT2	30 (23.1)	15 (15.0)	15 (50.0)	<0.0001	12 (18.5)	18 (27.7)	0.2979
	pT3 + pT4	100 (76.9)	85 (85.0)	15 (50.0)		53 (81.5)	47 (72.3)	
pN (n = 130)	pN0	54 (41.5)	34 (34.0)	20 (66.7)	0.0015	26 (40.0)	28 (43.1)	0.8588
	pN1-2	76 (58.5)	66 (66.0)	10 (33.3)		39 (60.0)	37 (56.9)	
cT (n = 30)	cT1-2	9 (31.0)	6 (27.3)	3 (42.9)	0.6424	5 (45.5)	4 (22.2)	0.2371
	cT3-4	20 (69.0)	16 (72.7)	4 (57.1)		6 (54.6)	14 (77.8)	
cN (n = 113)	cN0	68 (62.4)	50 (59.5)	18 (72.0)	0.2583	32 (57.1)	36 (67.9)	0.3229
	cN1-2	41 (37.6)	34 (40.5)	7 (28.0)		24 (42.9)	17 (32.1)	
No. LN collected (n = 130)	Median (min, max)	22 (4-73)	23.0 (6-67)	16.5 (4-73)	0.0842	23.0 (4-67)	18 (6-73)	0.1369
Tumour deposits (n = 108)	0	91 (84.3)	67 (79.8)	24 (100.0)	0.0117	42 (82.4)	49 (86.0)	0.792
	≥1	17 (15.7)	17 (20.2)	0 (0.0)		9 (17.7)	8 (14.0)	
Metastasis (n = 123)	cM0	86 (69.9)	63 (67.0)	23 (79.3)	0.2519	42 (68.8)	44 (71.0)	0.8457
	cM1	37 (30.1)	31 (33.0)	6 (20.7)		19 (31.2)	18 (29.0)	
Lymphatic invasion (n = 113)	L0	29 (25.7)	17 (19.3)	12 (48.0)	0.008	10 (18.5)	19 (32.2)	0.1312
	L1	84 (74.3)	71 (80.7)	13 (53.0)		44 (81.5)	40 (67.8)	
Venous invasion (n = 114)	V0	57 (50.0)	39 (44.3)	18 (69.2)	0.0433	26 (47.3)	31 (52.5)	0.708
	V1-2	57 (50.0)	49 (55.7)	8 (30.8)		29 (52.7)	28 (47.5)	
Perineural invasion (n = 111)	Pn0	100 (90.1)	75 (87.2)	25 (100.0)	0.0671	48 (90.6)	52 (89.7)	1.0
	Pn1	11 (9.1)	11 (12.8)	0 (0.0)		5 (9.4)	6 (10.3)	
Tumour grade (n = 130)	G1-2	85 (65.4)	66 (66.0)	19 (63.3)	0.8286	40 (61.5)	45 (69.2)	0.3566
	G3	45 (34.6)	34 (34.0)	11 (36.7)		25 (38.5)	20 (30.8)	
Postoperative therapy (n = 127)	None	88 (69.3)	65 (66.3)	23 (79.3)	0.2524	41 (64.1)	47 (74.6)	0.2491
	Yes	39 (30.7)	33 (33.7)	6 (20.7)		23 (36.0)	16 (25.4)	
MMR status (n = 117)	Proficient	99 (84.6)	77 (85.6)	22 (81.5)	0.5596	52 (89.7)	47 (79.7)	0.1996
	Deficient	18 (15.4)	13 (14.4)	5 (18.5)		6 (10.3)	12 (20.3)	
Survival time (n = 76)	Median (95% CI) months	73.5 (29-105)	53.1 (22.6-73.9)	Not reached	0.0026	26.4 (13.7-73.5)	105.4 (54.9-NE)	0.0053

### Statistics

The Pearson’s correlation coefficient (r) was used to determine the strength of the linear relationship between immune cell counts in the biopsy and resection. The inter-observer agreement was determined using the intraclass correlation coefficient (ICC), with values approaching 1.0 indicating improved agreement. Logistic regression analysis was performed to calculate the probability of lymph node metastasis as a function of the number of immune cells in the biopsy. Briefly, the probability p = e^(βo+β1x)^/(1- e^(βo+β1x)^), where βo and β1 are estimates of the logistic equation and x is the number of immune cells. In order to select unbiased cut-off scores for “low” and “high” immune cell counts, we used the 50th- or 75th-percentiles. The Chi-Square or Fisher’s Exact test was used, where appropriate. Student’s *T*-test was applied for assessment of mean age differences. Univariate survival time analysis was carried out using the log-rank test and differences were plotted with Kaplan-Meier curves. After verification of the proportional hazards assumption, Cox regression analysis was performed with “low” immune cell counts as baseline hazard of 1.0. Hazard ratios (HR) and 95% CI were used to determine the effect of each variable on outcome. Included in the analysis were pT, pN, cM and postoperative therapy. P-values <0.05 were considered statistically significant. No correction for multiple comparisons was performed [18]. All analyses were carried out using SAS V9.2 (SAS Institute, Cary, NC).

## Results

### T-cell infiltration in biopsies of colon and rectal cancer

Stromal CD8 T-cells were considerably more frequent (median cell count per HPF 37.6 cells) than intraepithelial CD8 infiltrates (median cell count per HPF 3.4 cells). For CD45RO, a similar pattern was observed (median cell count CD45ROs = 56.6 cells; CD45ROi = 4.4 cells).

### CD8+ and CD45RO + in the preoperative biopsy and impact on survival- univariable

**Figure 2:**
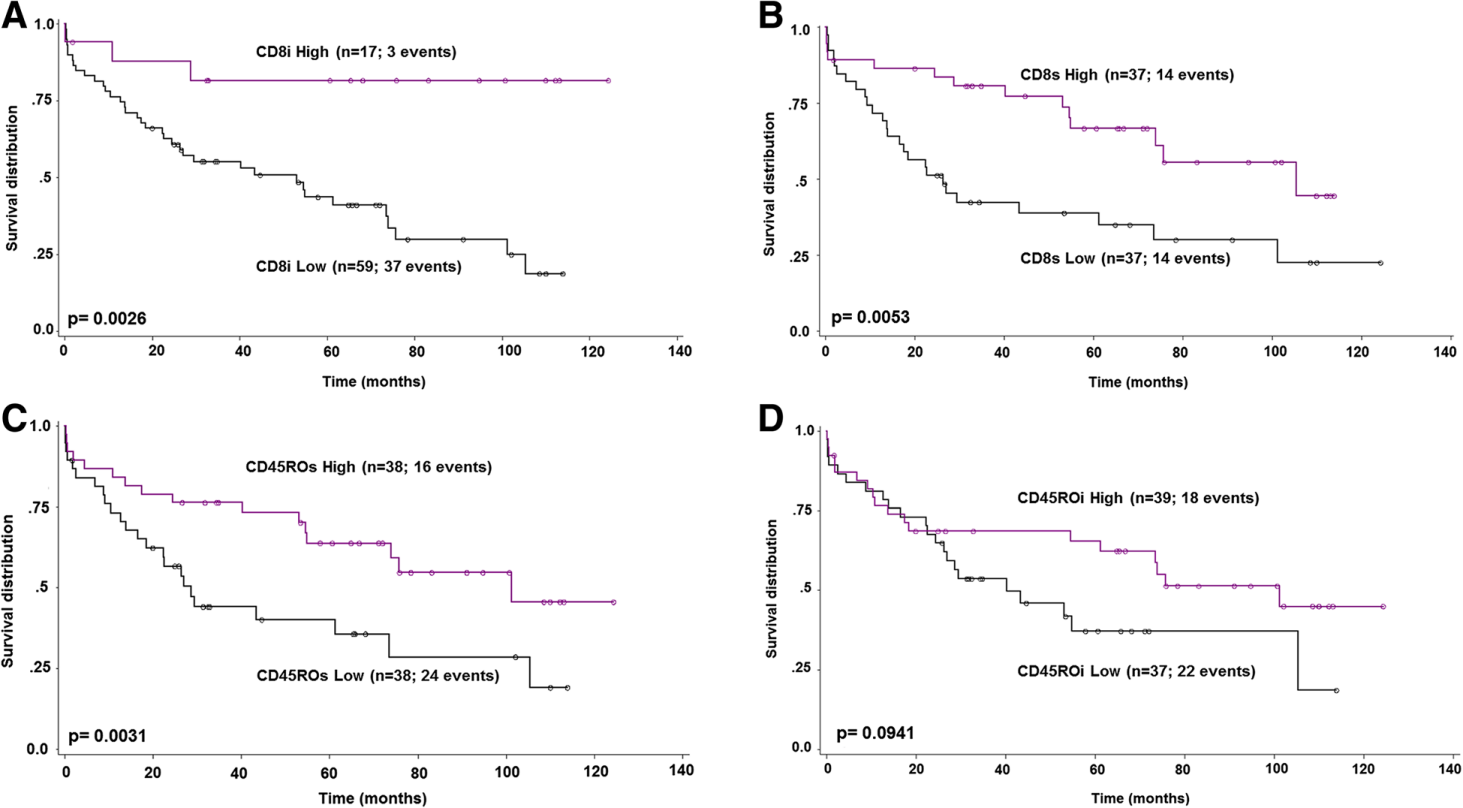
**Prognostic effects of CD8 and CD45RO T-cell infiltration in the pre-operative biopsy. A**, **B:** Strong infiltration by CD8 cells in the pre-operative biopsy predicted a significantly prolonged survival independent of stromal or intraepithelial location (CD8i: p = 0.0026; 75th-percentile as cut-off, >6.2 cells; CD8s: p = 0.0011; 50th percentile as cut-off, >37.6 cells). **C**, **D:** Patients with strong CD45ROs infiltration derived a significant survival benefit (CD45ROs: p = 0.0031; 50th-percentile as cut-off, >56.6 cells). No significant associations with survival time were observed for CD45ROi infiltrates (CD45ROi; p = 0.0941; 50th percentile as cut-off, > 3.4 cells).

CD8+ T-cell infiltration in the preoperative biopsy was significantly associated with favorable overall survival independent of stromal or intraepithelial T-cell location (CD8i, p = 0.0026; CD8s, p = 0.0053) (Figure 2A and B). For CD45RO, only stromal infiltrates showed a favorable association with survival time (p = 0.0031) (Figure 2C). No association with prognosis was observed for CD45ROi (p = 0.094) (Figure 2D).

### CD8+ and CD45RO + in the preoperative biopsy and impact on survival- multivariable

The strong correlation of a greater number of CD8+ and CD45RO+ T-cells with favorable prognosis is underlined in multivariable analysis (Table 2). The presence of CD8i in the biopsy maintained a significant favorable impact on survival after adjusting for the confounding effect of TNM-classification in the resection specimen (pT, pN, cM) and adjuvant therapy (HR = 0.55 (95% CI: 0.36-0.82); p = 0.0038). Similar findings were observed for CD8s (HR = 0.72 (95% CI: 0.57-0.9); p = 0.0049) and CD45ROs (HR = 0.76 (95% CI: 0.61-0.96); p = 0.0213). As determined in univariable analysis, CD45ROi was not associated with survival time in multivariable analysis.

**Table 2:** Multivariable analysis of CD8 and CD45RO with pT. pN, cM, and postoperative therapy

		**CD8i**		**CD8s**		**CD45ROi**		**CD45ROs**	
		**HR (95% CI)**	**P-value**	**HR (95% CI)**	**P-value**	**HR (95% CI)**	**P-value**	**HR (95% CI)**	**P-value**
Marker	Low	1.0	0.0038	1.0	0.0049	1.0	0.2644	1.0	0.0213
	High	0.55 (0.36-0.82)		0.72 (0.57-0.9)		0.88 (0.71-1.1)		0.76 (0.61-0.96)	
pT	pT1-2	1.0	0.9525	1.0	0.4854	1.0	0.3584	1.0	0.4897
	pT3-4	1.03 (0.39-2.75)		1.41 (0.54-3.67)		1.59 (0.6-4.3)		1.41 (0.53-3.72)	
pN	pN0	1.0	0.6523	1.0	0.7057	1.0	0.6596	1.0	0.3729
	pN1-2	0.82 (0.36-1.9)		0.85 (0.37-1.95)		0.83 (0.36-1.9)		0.69 (0.3-1.6)	
cM	cM0	1.0	0.0683	1.0	0.0485	1.0	0.1844	1.0	0.1348
	cM1	2.05 (0.95-4.4)		2.2 (1.01-4.81)		1.7 (0.78-3.7)		1.8 (0.83-3.9)	
Postoperative therapy	None	1.0	0.2672	1.0	0.2425	1.0	0.3805	1.0	0.4301
	Yes	0.63 (0.28-1.42)		0.63 (0.29-1.37)		0.7 (0.32-1.55)		0.73 (0.33-1.6)	

### Prediction of TNM-stage of the resection specimen based on the evaluation of CD8+ and CD45RO + immune infiltrates in the preoperative biopsy

Clinicopathological associations of CD8+ and CD45RO+ T-cell infiltrates are summarized in (Table 1 and Additional file 1: Table S1). Patients with weak CD8i infiltration in the biopsy frequently presented with locally advanced tumors at the time of resection (p < 0.0001). In fact, 85% (n = 85) of patients with low CD8i in the pre-operative biopsy (<6.2 cells on average per 5 HPF; 75th percentile) were diagnosed with pT3 or pT4 primary tumors. No associations were observed for CD8s, CD45ROi or CD45ROs. Radiographic T-staging was available for a subset of patients (n = 30). As compared to pathological staging, radiographic T-staging underestimated the anatomic extent of the primary tumor (31% cT1/2 as compared to 6.2% pT1/2). No correlation of T-cell infiltration with radiographic T-stage was observed.

**Figure 3:**
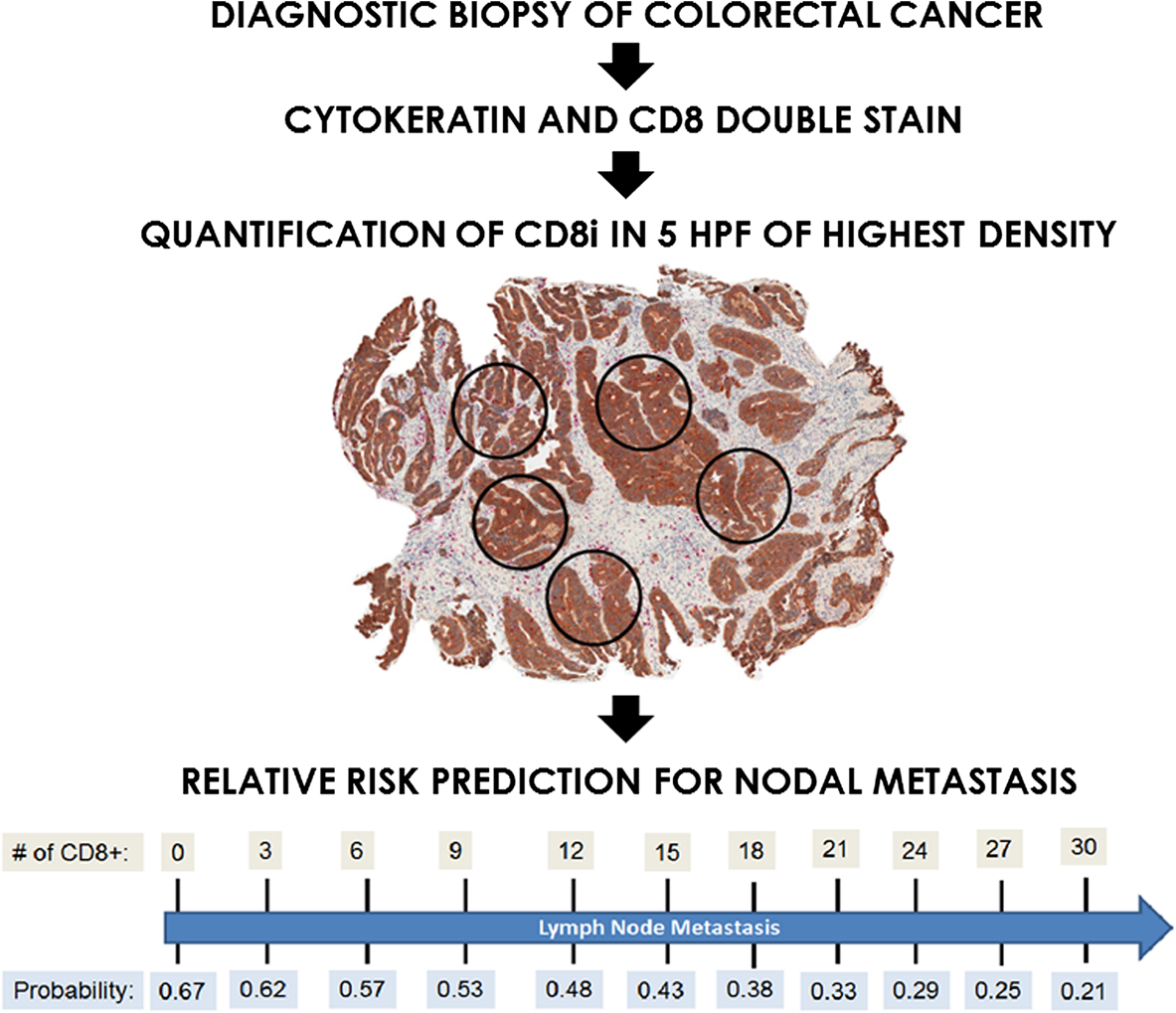
**Clinical application example.** Slides are first scanned under low magnification and regions of densest CD8i T-cell infiltrates are identified. CD8i T-cells infiltrates are then quantified in five HPF of highest density (HPF area 0.1886 mm^2^). The probability of lymph node metastasis can be determined based on a nomogram of CD8i counts. The fewer T-cells in the biopsy, the greater the risk of nodal metastasis.

A low CD8i count in the biopsy was significantly associated with detection of positive lymph nodes (p = 0.0015) in the resection specimen. Using the cut-off score of 6.2 cells, 66% (n = 66) of patients with low CD8i showed lymph node positivity in comparison to 33.3% (n = 10) patients with high CD8i counts. A probability scale for predicting the risk of lymph node metastasis as a function of CD8i infiltration is shown in (Figure 3). The more CD8i T-cells are observed, the lower the risk of nodal metastasis. For instance, a patient with absence of CD8i in the preoperative biopsy would have a 67% risk of nodal metastasis, which decreases to 35% in a patient with 20 CD8i in the biopsy and to only 21% when 30 CD8i are present. In practical application, this allows risk estimation of nodal metastasis based on the immunohistochemical staining of a single marker in biopsies of CRC.

Interestingly, the evaluation of CD45ROs provided additional discriminatory information for the identification of patients at high risk of nodal metastasis. A low CD45ROs count (<56.6 cells) was significantly associated with lymph node positivity (p = 0.0073) with 70.8% of patients with low CD45ROs counts (n = 46) presenting with nodal metastasis at the time of resection as compared to 46.2% (n = 30) with strong CD45ROs infiltration (Table 3).

**Table 3:** Combination of CD8i and CD45p for prediction of pN + (n = 130) shown are frequency (n) and column percent (%)

	**CD8i neg/CD45p neg**	**Only 1 positive**	**CD8i pos/CD45p pos**	**Total**	**P-value**
pN0	11 (21.6)	31 (49.2)	12 (75.0)	54	0.0002
pN+	40 (78.4)	32 (50.8)	4 (25.0)	76	
Total	51	63	16	130	

For 113 patients information on radiographic N-staging from pre-operative examinations was available (Table 1 and Additional file 1: Table S1). As compared to pathological staging, pre-operative radiographic staging appeared to underestimate the frequency of nodal metastasis (37.6% cN1-2 as compared to 58.5% pN1-2). Interestingly, no correlation of CD8+ or CD45RO + T-cell infiltration with radiographic N-staging was observed. A tendency towards a higher average number of CD8+ and CD45RO+ T-cell infiltrates was observed in biopsies from patients with absence of distant metastasis. However, the association between CD8+ or CD45RO+ T-cell infiltrates in the biopsy and absence of distant metastasis did not reach statistical significance.

### Additional prognostic factors

A greater number of CD8i T-cells in biopsy samples was strongly predictive of the absence of lymphatic vessel invasion (p = 0.008), venous vessel invasion (p = 0.433), and absence of tumor deposits (p = 0.0117) in the resection specimen. For CD45ROs, a significant association with absence of venous invasion (p = 0.0073) was determined. Further, CD45ROi infiltrates were more frequently observed in patients with mismatch-repair deficient CRC (p = 0.02). No association of CD8i, CD8s or CD45ROs infiltration with MMR-status was identified.

### Correlation between CD8 and CD45RO immune cell counts in biopsy and resections

Full tissue sections of primary tumors (n = 50) were evaluated for CD8s and CD45ROs infiltration and were compared to the matched biopsy on a case by case basis. The correlation coefficient for CD8s was r = 0.42 (p < 0.0001) suggesting a moderate positive relationship. For CD45ROs, only a fair and non-significant correlation between biopsy and resection was observed (r = 0.16; p = 0.2864).

### Inter-observer agreement of CD8 and CD45RO immune cell counts in the biopsy

The ICC was used to calculate the inter-observer agreement in 50 biopsy cases for CD8i, CD8s, CD45ROi, and CD45ROs, with values closer to 1.0 indicating stronger agreement. For CD8i and CD8s, the ICC was 0.95 and 0.75 indicating excellent and strong agreement, respectively. For CD45ROi and CD45ROs, the ICC values were 0.16 and 0.49 indicating only poor to moderate agreement.

## Discussion

Accurate prognostication is essential for choosing the optimal therapeutic approach for primary CRC. Based on the evaluation of CD8 and CD45RO infiltrates in biopsy specimens of colon and rectal cancers, we demonstrate prediction of patient survival, the anatomic extension of the primary tumor, the presence of positive lymph nodes, lymphovascular invasion, and tumor deposits. The evaluation of T-cell infiltrates based on immunohistochemistry is easy, fast and reproducible indicating usefulness in daily diagnostic practice. Taken together, these results may impact the treatment strategy for CRC patients in distinct clinical settings.

First, we identify a highly significant and independent association of CD8 and CD45ROs T-cell infiltrates in the preoperative biopsy with patient survival after resection of the primary tumor. This data is in consistence with several large retrospective clinical studies including robust multivariable analyses that have identified CD8 and CD45RO immune infiltration as central prognostic indicator in CRC resection specimens [9,19]. The current study now suggests that the prognostic power of immune infiltrates in CRC can already be harnessed in the preoperative setting. This information significantly extends the spectrum of available preoperative prognostic indicators based on biopsy material. In clinical practice, this may provide important prognostic information in patients receiving preoperative radiotherapy and chemotherapy. As immune infiltrates cannot be reliably assessed in these cases after resection, the preoperative biopsy is critical in providing prognostic information in these cases. Consequently, CRC patients with low in-situ immune infiltration may be considered a high risk group and may benefit from pre-operative therapy followed by surgical resection [20-22,16].

Second, we demonstrate that CD8i and CD45ROs immune infiltration in the biopsy allows prediction of nodal metastasis and tumor deposits in the preoperative setting. This may provide important information to the surgeon when deciding on the optimal strategic approach to a CRC patient. In the setting of early invasive CRC, patients with a low or absent infiltration by CD8 and CD45RO T-cells may best be managed by segmental resection due to the significantly increased risk of lymph node metastasis [23]. Low T-cell infiltration in biopsies could therefore expand the list of established high risk features in early CRC as detailed by Quirke and colleagues in the European guidelines for quality assurance: incomplete excision, poor differentiation, lymphatic invasion, tumor budding, and venous invasion [23]. To further develop this preliminary evidence, statistically robust studies should be performed to confirm absence of T-cell infiltration in the preoperative biopsy as a high risk feature for early stage patients.

In patients with locally advanced operable colon cancer, preoperative chemotherapy is an important line of development [22,24]. Accurate identification of nodal positive patients is essential for patient selection. Radiographic staging is standard of care in the pre-operative setting but sub-optimal sensitivity and specificity values for detection of nodal metastasis have been reported [25]. Computed tomography may only correctly identify positive lymph nodes in 45% of cases, and cut-off diameters for identification of positive lymph-nodes are a matter of debate [25,26]. In our study, radiographic staging of locoregional nodes significantly underestimated the frequency of nodal metastasis (37.6% cN1-2 as compared to 58.5% pN1-2). In this setting, assessment of T-cell infiltrates in biopsy specimens may provide additional valuable information on the relative risk for nodal metastasis.

Third, the preoperative estimation of tumor stage may also assist in planning the optimal surgical approach in locally advanced colon and rectal cancer. We demonstrate that patients with low CD8i infiltration in the biopsy are at high risk of transmurally invasive primary tumors with lymphovascular invasion, spread to local lymph nodes and formation of tumor deposits in the pericolic tissue. In colon cancer, extensive lymph node removal by resection in the mesocolonic plane should be advocated to reduce the relative risk of tumor recurrence and improve long term survival for these patients [27-29]. In rectal cancer, pre-operative staging has profound therapeutic implications. Early stage patients may be treated by radiotherapy alone while locally advanced or nodal-positive patients may be treated with chemotherapy or radiotherapy before resection [30]. In addition to radiographic staging, low immune infiltration may indicate the need for a risk-adapted therapeutic approach taking into consideration an elevated risk of lateral lymph node metastasis, deep tumor infiltration, and presence of tumor deposits.

The present study has several strengths. First, we provide a novel approach for prognostication of CRC patients in the preoperative setting based on a robust and well-established biomarker. The prognostic value of CD8 infiltration in primary CRC has been established in numerous large and statistically robust studies and this marker is included as central feature in the immunoscore consortium led by the Society for Immunotherapy of Cancer (SITC) [31]. Second, our analysis is based on a well-characterized cohort with complete follow-up including CRC patients of all stages. Third, this study is designed in accordance with the REMARK guidelines for tumor marker prognostic studies including assessment of inter-observer reproducibility [17]. Based on the superior reproducibility and prognostic value of CD8 over CD45RO as a biomarker, we provide a specific practical approach requiring immunostaining of a single slide for CD8 infiltrates followed by quantification of T-cell infiltrates in five HPF of highest density. CD8 infiltrates may also be efficiently evaluated using bioinformatics approaches saving time in the manual assessment of slides [9]. Consequently, evaluation of this single marker by immunohistochemistry in biopsies is a promising method of risk prediction in daily diagnostic practice.

Weaknesses of the study include the limited sample size involving only a relatively small cohort of rectal cancer cases. Cut off levels for T-cell infiltration may be influenced by the inherent characteristics of the study collective. Consequently, we recommend prospective investigation and external validation of the prognostic value of CD8+ and CD45RO + immune infiltrates in biopsy specimens on independent cohorts.

## Conclusions

Assessment of T-cell infiltration in preoperative biopsies provides additional independent prognostic information for CRC patients. Quantification of CD8i T-cell infiltrates may aid standard radiographic and clinical staging of the primary tumor and provide information on the relative risk for nodal metastasis before surgery. Evaluation of CD8i infiltration by immunohistochemistry is highly reproducible and shows superior potential as a prognostic marker in comparison to CD45RO. The assessment of CD8i T-cell infiltration in biopsies is recommended for prospective investigation.

### Ethics committee approval

The local ethics committee of the Insel University Hospital (16-03-12) approved this study.

## Abbreviations

CEA: Carcinoembryoenic antigen; CRC: Colorectal cancer; EMT: Epithelial-mesenchymal transition; HPF: High-power fields; HR: Hazard ratio; ICC: Intra-class correlation coefficient; ITB: Intra-tumoral budding; REMARK: REporting recommendations for tumor MARKer prognostic studies; SITC: Society for Immunotherapy of Cancer; TNM-stage: Tumor Node Metastasis stage.

## Competing interests

The authors have no relevant affiliations or financial involvement with any organization or entity with a financial interest in or financial conflict with the subject matter or materials discussed in the manuscript. This includes employment, consultancies, honoraria, stock ownership or options, expert testimony, grants or patents received or pending, or royalties.

## Authors’ contributions

VHK scored immunohistochemistry, reviewed cases, performed data interpretation and together with IZ drafted the manuscript; IZ performed data interpretation and statistical analysis and performed manuscript editing. AL reviewed cases and together with DI conceived the study and study design, and performed manuscript editing; HD reviewed cases and scored immunohistochemistry. MM, JA and JAG scored resection specimens and contributed to inter-observer assessment. MH, MDB, MB and BS obtained, reviewed and categorized clinical data. All authors read and approved the final manuscript.

## Supplementary Material

Additional file 1: Table S1 Association of CD45RO + cells (intra-and peri- tumoral) and clinicopathological data (n = 130). Click here for file

## References

[B1] Hanahan D Weinberg RA Hallmarks of cancer: the next generation Cell 2011 144 646 674 10.1016/j.cell.2011.02.013 21376230

[B2] Lugli A Karamitopoulou E Panayiotides I Karakitsos P Rallis G Peros G Iezzi G Spagnoli G Bihl M Terracciano L Zlobec I CD8+ lymphocytes/ tumour-budding index: an independent prognostic factor representing a 'pro-/anti-tumour' approach to tumour host interaction in colorectal cancer Br J Canc 2009 101 1382 1392 10.1038/sj.bjc.6605318 PMC2768462 19755986

[B3] Galon J Fridman WH Pages F The adaptive immunologic microenvironment in colorectal cancer: a novel perspective Canc Res 2007 67 1883 1886 10.1158/0008-5472.CAN-06-4806 17332313

[B4] De Craene B Berx G Regulatory networks defining EMT during cancer initiation and progression Nat Rev Canc 2013 13 97 110 10.1038/nrc3447 23344542

[B5] Prall F Tumour budding in colorectal carcinoma Histopathology 2007 50 151 162 10.1111/j.1365-2559.2006.02551.x 17204028

[B6] Hase K Shatney C Johnson D Trollope M Vierra M Prognostic value of tumor "budding" in patients with colorectal cancer Dis Colon Rectum 1993 36 627 635 10.1007/BF02238588 8348847

[B7] Wang LM Kevans D Mulcahy H O'Sullivan J Fennelly D Hyland J O'Donoghue D Sheahan K Tumor budding is a strong and reproducible prognostic marker in T3N0 colorectal cancer Am J Surg Pathol 2009 33 134 141 10.1097/PAS.0b013e318184cd55 18971777

[B8] Ueno H Mochizuki H Hashiguchi Y Shimazaki H Aida S Hase K Matsukuma S Kanai T Kurihara H Ozawa K Yoshimura K Bekku S Risk factors for an adverse outcome in early invasive colorectal carcinoma Gastroenterology 2004 127 385 394 10.1053/j.gastro.2004.04.022 15300569

[B9] Galon J Costes A Sanchez-Cabo F Kirilovsky A Mlecnik B Lagorce-Pages C Tosolini M Camus M Berger A Wind P Zinzindohoué F Bruneval P Cugnenc PH Trajanoski Z Fridman WH Pagès F Type, density, and location of immune cells within human colorectal tumors predict clinical outcome Science 2006 313 1960 1964 10.1126/science.1129139 17008531

[B10] Galon J Pages F Marincola FM Thurin M Trinchieri G Fox BA Gajewski TF Ascierto PA The immune score as a new possible approach for the classification of cancer J Transl Med 2012 10 1 10.1186/1479-5876-10-1 22214470 PMC3269368

[B11] Zlobec I Minoo P Terracciano L Baker K Lugli A Characterization of the immunological microenvironment of tumour buds and its impact on prognosis in mismatch repair-proficient and -deficient colorectal cancers Histopathology 2011 59 482 495 10.1111/j.1365-2559.2011.03975.x 22034888

[B12] Vayrynen JP Tuomisto A Klintrup K Makela J Karttunen TJ Makinen MJ Detailed analysis of inflammatory cell infiltration in colorectal cancer Br J Canc 2013 109 1839 1847 10.1038/bjc.2013.508 PMC3790164 24008661

[B13] Giger OT Comtesse SC Lugli A Zlobec I Kurrer MO Intra-tumoral budding in preoperative biopsy specimens predicts lymph node and distant metastasis in patients with colorectal cancer Mod Pathol 2012 25 1048 1053 10.1038/modpathol.2012.56 22481282

[B14] Rogers AC Gibbons D Hanly AM Hyland JM O'Connell PR Winter DC Sheahan K Prognostic significance of tumor budding in rectal cancer biopsies before neoadjuvant therapy Mod Pathol 2013 27 156 62 23887296 10.1038/modpathol.2013.124

[B15] Lugli A Vlajnic T Giger O Karamitopoulou E Patsouris ES Peros G Terracciano LM Zlobec I Intratumoral budding as a potential parameter of tumor progression in mismatch repair-proficient and mismatch repair-deficient colorectal cancer patients Hum Pathol 2011 42 1833 1840 10.1016/j.humpath.2011.02.010 21664647

[B16] Yasuda K Nirei T Sunami E Nagawa H Kitayama J Density of CD4(+) and CD8(+) T lymphocytes in biopsy samples can be a predictor of pathological response to chemoradiotherapy (CRT) for rectal cancer Radiat Oncol 2011 6 49 10.1186/1748-717X-6-49 21575175 PMC3120676

[B17] Altman DG McShane LM Sauerbrei W Taube SE Reporting recommendations for tumor marker prognostic studies (REMARK): explanation and elaboration BMC Med 2012 10 51 10.1186/1741-7015-10-51 22642691 PMC3362748

[B18] Perneger TV What's wrong with Bonferroni adjustments BMJ 1998 316 1236 1238 10.1136/bmj.316.7139.1236 9553006 PMC1112991

[B19] Fridman WH Pages F Sautes-Fridman C Galon J The immune contexture in human tumours: impact on clinical outcome Nat Rev Canc 2012 12 298 306 10.1038/nrc3245 22419253

[B20] Sebag-Montefiore D Stephens RJ Steele R Monson J Grieve R Khanna S Quirke P Couture J de Metz C Myint AS Bessell E Griffiths G Thompson LC Parmar M Preoperative radiotherapy versus selective postoperative chemoradiotherapy in patients with rectal cancer (MRC CR07 and NCIC-CTG C016): a multicentre, randomised trial Lancet 2009 373 811 820 10.1016/S0140-6736(09)60484-0 19269519 PMC2668947

[B21] Roh MS Colangelo LH O'Connell MJ Yothers G Deutsch M Allegra CJ Kahlenberg MS Baez-Diaz L Ursiny CS Petrelli NJ Wolmark N Preoperative multimodality therapy improves disease-free survival in patients with carcinoma of the rectum: NSABP R-03 J Clin Oncol 2009 27 5124 5130 10.1200/JCO.2009.22.0467 19770376 PMC2773471

[B22] Foxtrot Collaborative G Feasibility of preoperative chemotherapy for locally advanced, operable colon cancer: the pilot phase of a randomised controlled trial Lancet Oncol 2012 13 1152 1160 10.1016/S1470-2045(12)70348-0 23017669 PMC3488188

[B23] Quirke P Risio M Lambert R von Karsa L Vieth M Quality assurance in pathology in colorectal cancer screening and diagnosis-European recommendations Virchows Arch 2011 458 1 19 10.1007/s00428-010-0977-6 21061133 PMC3016207

[B24] Quasar Collaborative G Gray R Barnwell J McConkey C Hills RK Williams NS Kerr DJ Adjuvant chemotherapy versus observation in patients with colorectal cancer: a randomised study Lancet 2007 370 2020 2029 10.1016/S0140-6736(07)61866-2 18083404

[B25] Dewhurst C Rosen MP Blake MA Baker ME Cash BD Fidler JL Greene FL Hindman NM Jones B Katz DS Lalani T Miller FH Small WC Sudakoff GS Tulchinsky M Yaghmai V Yee J ACR appropriateness criteria pretreatment staging of colorectal cancer J Am Coll Radiol 2012 9 775 781 10.1016/j.jacr.2012.07.025 23122343

[B26] Thoeni RF Colorectal cancer. Radiologic staging Radiol Clin North Am 1997 35 457 485 9087214

[B27] Wong SL Lymph node counts and survival rates after resection for colon and rectal cancer Gastrointest Canc Res 2009 3 S33 S35 PMC2684729 19461921

[B28] West NP Hohenberger W Weber K Perrakis A Finan PJ Quirke P Complete mesocolic excision with central vascular ligation produces an oncologically superior specimen compared with standard surgery for carcinoma of the colon J Clin Oncol 2010 28 272 278 10.1200/JCO.2009.24.1448 19949013

[B29] West NP Kobayashi H Takahashi K Perrakis A Weber K Hohenberger W Sugihara K Quirke P Understanding optimal colonic cancer surgery: comparison of Japanese D3 resection and European complete mesocolic excision with central vascular ligation J Clin Oncol 2012 30 1763 1769 10.1200/JCO.2011.38.3992 22473170

[B30] Bosset JF Calais G Mineur L Maingon P Stojanovic-Rundic S Bensadoun RJ Bardet E Beny A Ollier JC Bolla M Marchal D Van Laethem JL Klein V Giralt J Clavère P Glanzmann C Cellier P Collette L EORTC Radiation Oncology Group Fluorouracil-based adjuvant chemotherapy after preoperative chemoradiotherapy in rectal cancer: long-term results of the EORTC 22921 randomised study Lancet Oncol 2014 15 184 190 10.1016/S1470-2045(13)70599-0 24440473

[B31] Galon J Pages F Marincola FM Angell HK Thurin M Lugli A Zlobec I Berger A Bifulco C Botti G Tatangelo F Britten CM Kreiter S Chouchane L Delrio P Arndt H Asslaber M Maio M Masucci GV Mihm M Vidal-Vanaclocha F Allison JP Gnjatic S Hakansson L Huber C Singh-Jasuja H Ottensmeier C Zwierzina H Laghi L Grizzi F Cancer classification using the immunoscore: a worldwide task force J Transl Med 2012 10 205 10.1186/1479-5876-10-205 23034130 PMC3554496

