# Supplementary material for: CD8/CD45RO T-cell infiltration in endoscopic biopsies of colorectal cancer predicts nodal metastasis and survival
Source: J Transl Med. 2014 Mar 29;12:81. doi: 10.1186/1479-5876-12-81 (PMC4022053; doi:10.1186/1479-5876-12-81)
Supplement: Additional file 1: Table S1 — Association of CD45RO + cells (intra-and peri- tumoral) and clinicopathological data (n = 130). [file 1479-5876-12-81-S1.doc]

**Additional file 1: Table S1**: Association of CD45RO+ cells (intra-and peri- tumoral) and clinicopathological data (n=130)

|  |  | CD45ROi N (%) | |  | CD45ROs N (%) | |  |
| --- | --- | --- | --- | --- | --- | --- | --- |
|  |  | Low | High | P-value | Low | High | P-value |
| Age (years) | Median (min, max) | 72.6 (30-90) | 71.6 (46-91) | 0.6004 | 70.9 (30-90) | 73.8 (30-91) | 0.5011 |
|  |  |  |  |  |  |  |  |
| Gender | Male | 38 (58.5) | 42 (64.6) | 0.5888 | 37 (56.9) | 43 (66.2) | 0.2794 |
|  | Female | 27 (41.5) | 23 (35.4) |  | 28 (43.1) | 22 (33.9) |  |
|  |  |  |  |  |  |  |  |
| Histological subtype | Non-mucinous | 54 (83.1) | 52 (80.0) | 0.8216 | 51 (78.5) | 55 (84.6) | 0.4983 |
|  | Mucinous | 11 (16.9) | 13 (20.0) |  | 14 (21.5) | 10 (15.4) |  |
|  |  |  |  |  |  |  |  |
| Tumour location | Left | 23 (35.4) | 20 (31.3) | 0.4301 | 19 (29.7) | 24 (36.9) | 0.5092 |
|  | Rectum | 12 (18.5) | 18 (28.1) |  | 14 (21.9) | 16 (24.6) |  |
|  | Right | 30 (46.2) | 26 (40.6) |  | 31 (48.4) | 25 (38.5) |  |
|  |  |  |  |  |  |  |  |
| pT | pT1+pT2 | 13 (20.0) | 17 (26.2) | 0.5328 | 13 (20.0) | 17 (26.2) | 0.405 |
|  | pT3+pT4 | 52 (80.0) | 48 (73.9) |  | 52 (80.0) | 48 (73.9) |  |
|  |  |  |  |  |  |  |  |
| pN | pN0 | 24 (36.9) | 30 (46.2) | 0.2856 | 19 (29.2) | 35 (53.9) | 0.0073 |
|  | pN1-2 | 41 (63.1) | 35 (53.9) |  | 46 (70.8) | 30 (46.2) |  |
|  |  |  |  |  |  |  |  |
| *cT (n=29)* | *cT1-2* | *3 (23.1)* | *6 (37.5)* | *0.4543* | *3 (22.1)* | *6 (37.5)* | *0.4543* |
|  | *cT3-4* | *10 (76.9)* | *10 (62.5)* |  | *10 (76.9)* | *10 (62.5)* |  |
|  |  |  |  |  |  |  |  |
| *cN (n=109)* | *cN0* | *38 (67.9)* | *30 (56.6)* | *0.242* | *32 (59.3)* | *36 (65.5)* | *0.5563* |
|  | *cN1-2* | *18 (32.1)* | *23 (43.4)* |  | *22 (40.7)* | *19 (34.6)* |  |
|  |  |  |  |  |  |  |  |
| Tumour deposits | 0 | 47 (82.5) | 44 (86.3) | 0.6095 | 47 (81.0) | 44 (88.0) | 0.3216 |
|  | ≥1 | 10 (17.5() | 7 (13.7) |  | 11 (19.0) | 6 (12.0) |  |
|  |  |  |  |  |  |  |  |
| Metastasis | cM0 | 42 (68.9) | 44 (71.0) | 0.8457 | 40 (66.7) | 46 (73.0) | 0.4428 |
|  | cM1 | 19 (31.2) | 18 (29.0) |  | 20 (33.3) | 17 (27.0) |  |
|  |  |  |  |  |  |  |  |
| Lymphatic invasion | L0 | 12 (20.3) | 17 (31.5) | 0.2006 | 12 (20.0) | 17 (32.1) | 0.1953 |
|  | L1 | 47 (79.7) | 37 (68.5) |  | 48 (80.0) | 36 (67.9) |  |
|  |  |  |  |  |  |  |  |
| Venous invasion | V0 | 33 (55.0) | 24 (44.4) | 0.3484 | 22 (36.1) | 35 (66.0) | 0.0025 |
|  | V1-2 | 27 (45.0) | 30 (55.6) |  | 39 (63.9) | 18 (34.0) |  |
|  |  |  |  |  |  |  |  |
| Perineural invasion | Pn0 | 54 (91.5) | 46 (88.5) | 0.5898 | 53 (89.8) | 47 (90.4) | 1.0 |
|  | Pn1 | 5 (8.5) | 6 (11.5) |  | 6 (10.2) | 5 (9.6) |  |
|  |  |  |  |  |  |  |  |
| Tumour grade | G1-2 | 44 (67.7) | 41 (63.1) | 0.5802 | 38 (58.5) | 47 (72.3) | 0.1398 |
|  | G3 | 21 (32.3) | 24 (36.9) |  | 27 (41.5) | 18 (27.7) |  |
|  |  |  |  |  |  |  |  |
| Postoperative therapy | None | 43 (68.3) | 45 (70.3) | 0.8489 | 42 (66.7) | 46 (71.9) | 0.5676 |
|  | Yes | 20 (31.8) | 19 (29.7) |  | 21 (33.3) | 18 (28.1) |  |
|  |  |  |  |  |  |  |  |
| MMR status | Proficient | 53 (93.0) | 46 (76.7) | 0.02 | 51 (86.4) | 48 (82.8) | 0.6173 |
|  | Deficient | 4 (7.0) | 14 (23.3) |  | 8 (13.6) | 10 (17.2) |  |
|  |  |  |  |  |  |  |  |
